# Experiences with out-patient hospital service utilisation among older persons in the Asante Akyem North District- Ghana

**DOI:** 10.1186/s12913-017-2604-6

**Published:** 2017-09-13

**Authors:** Jonathan Bayuo

**Affiliations:** Department of Nursing, Faculty of Health and Medical Sciences, Presbyterian University College, P.O. BOX 42, Agogo, Ghana

## Abstract

**Background:**

Though ageing is not a disease, it has been associated with the occurrence of conditions which require health service utilisation. Ghana’s population is characterised by a steady growth in the number of older adults and previous studies have noted limited levels regarding utilisation by older persons.

**Methods:**

Thus, this study utilised a qualitative approach to explore older persons’ experiences regarding out-patient hospital service utilisation in the Asante Akyem North District of Ghana. The aim was to generate findings that will guide future policies. Sixteen semi-structured interviews were conducted and thematic analysis executed. The Andersen’s Behavioural Model was used as a guiding framework.

**Results:**

Medical condition was noted to characterise the need component of utilisation. Also, perceived effects of ageing, beliefs and past health status predisposed an older person to utilise available services. Beliefs were noted to make an older person utilise either orthodox or herbal services. Despite these, family support (in the form of financial assistance), accessibility (health facility, health professional, medication and information) and health care costs either enabled or prevented an older person from utilising services. Despite the existence of the National Health Insurance Scheme, health care costs are high and that delayed utilisation or made others avoid the services altogether. The care processes were noted to be cumbersome and involved long hours; though these features were noted to be absent whilst utilising traditional medicine services and this provides an avenue for further research in assessing patient outcomes associated with traditional medicine usage. These findings might be contributing factors to why other studies identified limited usage of health services among older persons in Ghana.

**Conclusion:**

Though older persons in the district may feel the need to utilise health services on outpatient basis, the enabling factors (notably finance) appeared to be a driving force to actual utilisation. Thus, more innovative health care financing strategies are needed to enhance the coverage of health services for older persons in the district.

## Background

The world’s population of older persons has been growing for centuries, but the pace of growth has accelerated in recent times. The global population of persons aged 65 years or older was estimated at 461 million in 2004, an increase of 10.3 million since 2003. In 2010, the World Health Organisation [1] noted that an estimated 524 million people were aged 65 years or older and that formed 8% of the world’s population. Projections suggest that the annual increase will continue to exceed 10 million over the next decade with an estimated monthly rise of more than 850,000 [2]. This global picture may suggest that the number of older persons is likely to outnumber the number of children below age of five years [2].

Kinsella & Philips [3] have argued that while the population of more developed countries have been ageing for almost over a century now, this process began recently in developing countries. Inasmuch as developing countries may triumph over an increasing number of persons growing to advanced ages, the reality of meeting the needs of older persons’ in these places requires attention. This is based on the assertion that most policies in developing countries have focused on children and mothers and it will be a great challenge to re-focus attention on older persons [4].

Within the Ghanaian context, issues relating to older persons gained governmental attention in the late 1980’s when steady increment in the number older persons was noted [5]. Thus, inasmuch as Ghana’s population appears youthful, the absolute number of older persons appears to be increasing at a steady rate and this has been projected to increase further [5].

The process of ageing, though not a disease may be associated with various disease conditions which may warrant the utilisation of health services [6]. In terms of meeting the health needs of older persons, the Ghanaian health system has been described as “weak and resource-constrained” [7]. Also, the current hospital system has been noted to be overly associated with acute care; though older persons may be affected with chronic ailments [8]. Consequently, the Ghana Public Health Association (GPHA) has expressed concerns regarding poor utilisation of health care services among older persons and inadequate access to specialised health services [9]. In similar lines, Tawiah [10] has also argued that the greatest challenge of old age is to win the war against degenerative chronic conditions affecting older persons. To this end, the Ghana Statistical Services (GSS) and the Ghana Public Health Association have recommended health care and health policy reforms to meet the needs of older persons [7, 9].

In pursuing these reforms, GSS has called for “re-integration of older persons into the process so as to enable them contribute to their own well-being” [7]. This may mean that there is a need to capture their experiences with current health services as these may serve as a basis to understanding how they feel about current services and what their expectations are in relation to health care service utilisation [11]. It is worth noting that some quantitative studies have produced evidence regarding limited levels of health service utilisation among older persons in Ghana (example Exavery et al., [12]), however, a description of their experiences whilst utilising health care remains blurred. Thus it is believed that a thorough description of their experiences can establish an understanding of how well the outpatient hospital services are meeting their needs and how they feel about the services as these can guide future reforms and policies [11].

### The Ghanaian health care and health insurance system

The approach to health care in Ghana represents a mixture of preventive and curative services. Before 1996, health care delivery was the sole responsibility of the Ministry of Health and its services were basically oriented towards curative services. However in 1996, an act of parliament (ACT 525) established the development of Ghana Health Service which appeared to be more oriented towards preventive services; though focus currently remains heavily on maternal and child health. Healthcare services are provided by hospitals, health centres, maternity homes and chemical shops. However, the well-resourced hospitals or tertiary health facilities are located mostly in the urban places though most of the older persons are in rural areas. In addition, there exist traditional medical services at various locations in the country.

As part of the government’s effort to ensure equitable access to health in Ghana, it introduced the National Health Insurance Scheme (NHIS) into the health system. The primary goal of the scheme is to increase affordability and utilisation of drugs and health services in general, and among the poor and most vulnerable populations in Ghana. The NHIS is financed from four main sources: a value added tax on goods and services, a portion of social security taxes from formal sector workers, individual premiums, and miscellaneous other funds from investment returns, Parliament, or donors. The 2.5% tax on goods and services, called the National Health Insurance Levy (NHIL), is by far the largest source, comprising about 70% of revenues. Social security taxes account for an additional 23%, premiums for about 5%, and other funds for the remaining 2 %. The NHIS covers outpatient services, including diagnostic testing and surgeries such as hernia repair; some in-patient services, including specialist care, most surgeries, and hospital accommodation (general ward); oral health treatments; all maternity care services, including Caesarean deliveries; emergency care; and, finally, all drugs on the centrally-established National Health Insurance Authority (NHIA) Medicines List (NHIA webpage). In order to be enrolled, a Ghanaian citizen is expected to pay a registration fee and a premium (has to be renewed on a yearly basis). However, persons aged 70 years and above, core poor and pregnant women are exempted from paying premiums but must pay the registration fee prior to enrolment. Aside the NHIS, there are various private insurance schemes available to persons in the country.

### Theoretical framework

**Fig. 1:**
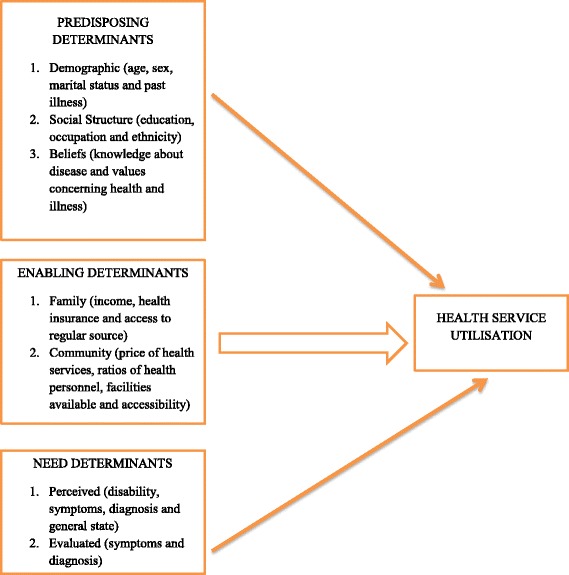
Health Service Utilisation Model (Andersen [15])

The Andersen’s Behavioural Model of Health Care Utilisation was developed in the late 1960’s to assist in understanding why people use or do not use health care services and to measure equitable access to health care [13]. Health care utilisation is seen as an essential stride towards illness management, prevention of diseases and treatment [14]. Health care utilisation has been defined by Andersen’s model as interplay of predisposing, enabling and need determinants. Thus, an individual’s access to and use of health services is a function of these three determinants or characteristics. A simplified version of the model is presented as Fig. 1 below. Though studies have specified the usefulness of the model, it has been indicated that it offers better explanation for discretionary health behaviours (outpatient care services) than non-discretionary health behaviours such as inpatient care [15]. Similarly, the model has been noted not to be sensitive to the diverse cultural and structural barriers in healthcare among minority groups. Thus, Andersen [15] has suggested the need for careful integration of cultural and structural variables into the model so as to enable the model provide explanations regarding health service utilisation among minority groups. Furthermore, the model offers flexibility in understanding health behaviours and can be applied to the current study as it focused on outpatient service utilisation in the hospital [15]. The model is shown as Fig. 1.

## Methods

The aim of this study was to explore and describe the experiences of older persons regarding outpatient service utilisation in Asante Akyem North District.

### Study design

As this study was oriented towards exploring and describing older persons’ experiences with health service utilisation, an in-depth descriptive qualitative approach was appropriate [16]. Waldrop et al., [17] have noted that the in-depth qualitative approach is appropriate to provide textual descriptions of older persons’ experiences and useful as health service utilisation experiences among older persons appear to have been minimally explored.

### Setting

The study was conducted within the Asante Akyem North District of the Republic of Ghana; specifically, the Agogo Presbyterian Hospital (a quasi-governmental health care facility). The hospital was selected as it is the largest health facility in the district and is utilised by persons of all ages. The services offered include both preventive and curative care. Curative care services have been divided into medical, surgical, obstetrics, paediatrics and emergency services. Older persons most often utilise medical and surgical care services on either outpatient or inpatient basis [7].

### Participant recruitment

The purposive sampling approach which involved specifically recruiting persons aged 50 years and over [16] was utilised in this study. Thus, after obtaining ethical clearance at the University of Southampton and the Agogo Presbyterian Hospital, information was sent to the Out-Patient Department (OPD) of the hospital to inform staff about the study. The researcher was provided a seat at the reception. After older persons had been attended to by the physician and were about to leave the hospital, they were met at the exit of the OPD reception by the researcher in order to discuss the study and invite them to participate. The discussions were carried out in the “Akan” dialect (a Ghanaian Language). The contact numbers of older persons who were interested in partaking in the study were obtained from them. After two days of purposively recruiting sixteen (16) older persons, phone calls were carried out from the third day and appointments were scheduled with participants at their own convenience and venue. The medical staffs were informed that in case data saturation was not achieved after interviewing the sixteenth person, a follow up recruitment would take place. However by the end of the sixteenth interview, data saturation was reached as no new information was noted [16]. As no new information was noted after this, the researcher did not return to the hospital to recruit more participants. Moreover as the study had to be completed within a stipulated time frame, the researcher proceeded to continue with data analysis.

### Data collection

As the study aimed to understand and describe older persons’ experiences regarding health service utilisation, the words and non-verbal language cues expressed by participants would be helpful. Thus, an interview was appropriate [16]. The interview approach was utilised as it was easier to schedule a meeting with one older person at a time as compared to focus group discussion and this allowed participants to express themselves in the presence of only the researcher [18]. Mason [19] has noted that semi-structured interviews provided deeper and rounded explanations. To this end, an interview guide was developed to obtain data from older persons using a semi-structured approach [20]. Before undertaking the interviews, the guide was piloted among three older persons who were conveniently recruited in the district. However, the findings from those interviews were not included in the actual study. The interviews took place at the participants’ home with minimal distraction. Prior to scheduling interviews, an initial discussion had been commenced with participants at the point of recruitment. Thus, participants were familiar with the researcher even before data collection commenced. Upon arrival at the interview venue, further explanations were offered regarding the study and participants were allowed to ask questions. Permission was sought to have the interview recorded. As participants spoke about their experiences, facial expressions were noted in a field diary. As the interview continued, intermittent breaks were provided to enable participants to take water. The interviews lasted between 49 to 65 min. The interview commenced with asking participants how they have been faring and this was followed by obtaining socio-demographic data. Participants were then asked what they perceived as their health needs and followed with how they felt with regards to utilising OPD services in the hospital. Also, participants were asked to describe how well the services were meeting their needs. As the interview proceeded, probes were used to enable in-depth exploration of their experiences. In some instances, an iterative mode of questioning was used to ensure that participants were consistent with their responses [16]. Leading questions and medical jargons were avoided throughout the interview process. At the end of the interview, participants were thanked and informed that all recordings would be transcribed to English and emerging themes discussed with them (as interviews were conducted in the “Akan” dialect). At a later day, a second interview was held with each participant. Feedback was obtained from each participant which further shaped the findings as some “Akan” terms were clarified and re-considered in the analysis process.

### Data management and analysis

In order to provide an in-depth description of older persons’ experiences with outpatient service utilisation, thematic analysis was used to generate themes from the data obtained [16]. Thematic analysis involved discovering, interpreting and reporting patterns and clusters of meaning within the data [18, 16]. This required working systematically through the transcribed texts and identifying themes that are progressively integrated into higher-order key themes in relation to the research questions [16]. Transcribed data were entered into MS word and exported to NVivo version 10. The analysis proceeded with an understanding of the Health Service Utilisation Model and findings offered support for the model.

### Methodological rigour

Participant validation and prolonged contact with participants helped to ensure that descriptions represented their experiences.

### Ethical considerations

Prior to commencement of the study, ethical clearance was sought and obtained from the Research & Governance Unit of the University of Southampton. Thereafter, the study was registered at the Agogo Presbyterian Hospital. Approval and clearance was then obtained at the hospital prior to commencement of the study. After recruitment, participants were provided with two consent forms to either thumbprint or sign.

## Results

### Socio-demographic characteristics of participants

The process of purpose sampling resulted in recruiting older persons aged 50 years or more for the study. A total of sixteen (16) older persons participated in the study. Details of socio-demographic features are presented in Table 1. The majority of the participants are within the ages of 50 to 59 years (*n* = 6). A greater number of older persons in the study are married (*n* = 9) and are Christians (*n* = 11).

**Table 1:** Socio-demographic features of participants

		50–59 years	60–69 years	70–79 years	80+
Age		6	5	3	2
Marital status	Married	5	3	1	–
	Single	–	1	–	–
	Widowed	1	1	2	2
	TOTAL	6	5	3	2
Religious affiliation	Christian	3	4	3	1
	Islam	2	1	–	1
	Traditional	1	–	–	0
	TOTAL	6	5	3	2
Gender	Male	2	3	0	1
	Female	4	2	3	1
	TOTAL	6	5	3	2

(Source: Field Data, 2016)

### Experiences with health service utilisation

Health service utilisation has been defined by Andersen’s behavioural model as interplay of needs, predisposing and enabling determinants. This section discusses themes associated with each determinant.

#### Need themes

These determinants specify the reason for seeking healthcare. In this study, medical condition (symptoms and diagnosis) and professional evaluation were identified as determinants that made older persons seek and utilise health care services.

##### Medical condition

From the analysis, the symptoms associated with a particular condition made an older person seek and utilise health care services. These symptoms appeared to interfere with the older person’s way of usual activities and as such decided to seek medical attention. For some participants, utilising hospital services occurred almost immediately; whilst others tried some home remedies before seeking attention at the hospital. The perceived severity of the symptom was either based on the intensity of the symptoms as experienced by participants or consequences of failing to act on initial symptoms experienced. In some instances, older persons had some knowledge about the complications that may result or had heard others talk about similar symptoms and that made them consider using hospital services:

*“I had been coughing for three days and I was unable to sleep well but I thought it would pass soon but it did not. I tried lime and honey at home but it just did not work so I went to the hospital. The cough was unbearable and it was disturbing everyone in the room when we slept at night [cups chin in the palm]” (Male, 60–69 years)*

*“I hardly visited the hospital until I started urinating too often which disturbed me a lot. If I continued like that I knew all the water in me would get finished so I had to go to the hospital and see the doctor for help” (Female, 70-79 years)*

##### Professional evaluation

Subsequently, as older persons felt the need to seek health care, they were evaluated by a health care provider and that made it necessary for an older person to continue using health services. In this regard, participants were informed of the need to attend regular reviews by physicians. This was in the form of monthly or bi-monthly medical appointment to assess progress made, carry out laboratory tests, change or refill medications. Thus, been informed by the physician of the need for reviews appeared to have created a need for continuous outpatient visits:

*“I used to go the hospital once every month but it has been changed to once every two months. I get my* blood pressure drugs for sixty days*” (Female, 50–59 years)*

*“They did so many things and the doctor said I should come there every month for check-up but now, I see my doctor once every two months for him to assess me and see if there is any problem. I get medications too” (Male, 83 years)*

#### Predisposing themes

The themes noted in this section include perceived effects of ageing, beliefs and past health care experiences.

##### Perceived effects of ageing

Participants verbalised the need to seek various forms of health care. A theme identified was that participants believed that increasing age predisposed them to utilise health services to a greater extent as they got ill often or experienced some physical symptoms which caused discomfort. Thus, participants indicated advancing age was associated with diminishing physical health which needed health care. Also, advancing age was noted to be associated with physical symptoms such as joint pains and the existence of chronic conditions such as diabetes that made the need for health care usage important:

*“Well as one grows older, things change. Now I can see that my strength has reduced and I am unable to work as I do. When I walk for some few metres……. [pauses for a while] I am tired and feel pains in my calf. It is not like it is going to change because I know I am growing older with all the pain at my back and legs” (Male, 60–69 years)*

*“I wake up with pain every morning and have to come to the hospital because of it. I can really feel I am growing old with all these pains [sighs heavily]” (Female, 70-79 years)*

Despite the symptoms and conditions associated with advancing age, participants felt the need to maintain good health as they grew older and that created the likelihood of utilising outpatient hospital services even though some participants noted that it was difficult maintaining good health especially as one advanced in age. The difficulty associated with maintaining good health in older age was noted to be associated with the nature of the symptoms which appeared to be chronic and that made participants compare their health status in their youthful years with their current status. Despite this, it was identified that the difficulty in maintaining good health by themselves predisposed older persons to utilise health care as they desired complete recovery. This desire served as a predisposing factor that made older person require continuous contact with health care services:

*“At my age, I want to remain strong though it is difficult for me because I am not getting any younger. My health needs is all about those things that make me feel fit and strong. I want my blood pressure to always remain okay so that I do not experience any headaches in the evening when I sleep. It has been difficult for me though I try my best and I want to be completely well from these pains and high blood pressure” (Male, 60–69 years)*

“*When I was young, I did not experience this but I now have it and no matter what I do, I still feel weak at times and as for the pain, it is there always. I walk short distances and I have to wait for a while.” (Female, 60-69 years)*

##### Beliefs

Aside perceived effects of ageing, beliefs were also noted to predispose older persons to utilise health care services. However, these beliefs predisposed older persons to either utilise orthodox or traditional medical services. In this regard, if an older person believed the aetiology of their illness was due to pathological process, they sought orthodox service. However, if the older person believed the disease was associated with supernatural aetiology, they sought help from the traditional practitioners in addition to the orthodox service they received. Despite these variations, all participants believed it was necessary to maintain good health as they grew older and they valued it as such:

*“I think this sickness is coming along with my old age so I come to the hospital because they can be of help…… I don’t really believe in those stories where people say there is a curse or something. It is a disease and I have to go to the place where they treat it.” (Female, 60–69 years)*

*“Ah…………….I think it was because I offended some people in the past that is why I got that sugar disease [diabetes] so I visit the herbalist for assistance in addition to what the doctor will give me at the hospital” (Female, 50-59 years)*

##### Past health care experiences

It was noted that past experiences with health care services served as a determinant that allowed them to use that service again. This was associated with how satisfied they were with previous utilisation. Older persons were able to compare services of various health care facilities and based on their past experiences, they opted for a facility they felt more comfortable with:

*“I have been at the hospital three years now and in the past, anytime I felt sick I came here so I feel more comfortable been there; I know most of the people there too so I get comfortable with them as well” (Male, 60–69 years)*

*“Even though I spend more time waiting there, the Government hospital at Konongo is worse and it is terrible there so I prefer traveling here to see a doctor”. (Male, 70-79 years)*

#### Enabling themes

The themes noted here are family influence and support, accessibility and health care costs.

##### Family influence and support

In the presence of an illness, influence and support from close family members served as enabling determinants. Family influence was evident as some older children encouraged their parents to utilise available health services. Influence of older children was also reflected in reminding their parents of medical appointments and this allowed participants to keep track of when they had to be present at the hospital. Aside adult children, spouses were noted to be influential in enabling older persons utilise health care services: Support offered by adult children and spouses also comprised of financial support as that helped older persons pay for costs of health care utilisation:

*“It is my daughter who told me to go to the hospital because they have medications to help me” (Female, 70–79 years)*

*“Some time ago, I hardly visited the hospital until I started urinating too often. I did not want to come here because I will spend the entire day here until my daughter talked me into coming” (Male, 50-59 years)*

*“When my husband was alive, he occasionally came with me and paid the bills but now that he has died, I come alone and it is sometimes a problem for me especially when I do not have money” (Female, 70-79 years)*

##### Accessibility

Aside family support, it was noted that easy accessibility to health care services was identified as a key issue and appeared to dominate most part of the interview. Accessibility represented how easy an older person could reach or come into contact with the health care facility. For some participants, the hospital or other health care facilities were within immediate reach but other participants had to travel from other places to visit the hospital. Those who stayed close to the hospital most often utilised services as soon as they felt the need to and had the available resources. However, for older persons who stayed far from the hospital, pharmacy shops were the first point of call in case it became necessary to utilise health services. However, if no positive response is noted after utilising pharmacy services, the older person proceeded to the hospital at a later day and this implied leaving the house early and having extra money for transportation. The choice of transportation to the health facility depended on how much money an older person had and how fast they hope to arrive at their destination. Thus, financial constraints implied that the older persons would not be able to visit the health facility. In other instances, participants did not turn up for medical reviews if they noted that they would arrive at the hospital late which implied been seen by a health professional late. In these cases, participants remained home. Aside participants staying at home with their symptoms, some utilised traditional medicine as it was readily accessible than the orthodox services and participants did not have to travel far. Thus, in this instance herbal preparations served as substitute and were utilised because they were within easy reach:

*“I go to the pharmacy shop most often because it is close by and later come to the hospital if I still do not feel any better” (Male, 50–59 years)*

*“I leave the house very early in order to arrive here on time and see the doctor because if I come late, I am assured of getting back home late.” (Female, 60-69 years)*

*“Sometimes I do not have money for transport so I stay back home till I am able to raise some cash otherwise I will not go to the hospital. Even if I have money and time is past 8am, I will not go to the hospital because if I do I will come home very late. So I manage and live with the pain till I am able to wake up early enough and come and queue here to be seen by the doctor” (female, 60-69 years)*

*“The herbalist is just at the community centre close to my house so I can just walk there and take some herbs, boil them and drink twice a week” (Female, 60-69 years)*

Accessibility also represented how easy an older person came into contact with a health professional when they arrived at the health care facility to have their health care needs addressed. Participants had to go through various processes before been seen by a health care provider and that required they leave their various homes early to queue at the hospital. Thus, coming to the hospital was noted to be characterised by long waiting times, and cumbersome with several processes for older persons:

*“I have to wake up early and come and drop my card at the entrance. If I come late, I will leave late so I come early so that at least I can still go and farm when I leave the hospital.” (Female, 80 years)*

*“I have to wait long hours. After I have seen the doctor and I will be asked to take my blood to the lab for investigation. I will wait for the results and I can spend all morning. It is too long for me because I will have to join another queue to see the doctor with my results and another queue to pay and collect my medication.” (Male, 70-79 years)*

This identified problem was further compounded by inadequate sitting space which implied that some clients had to stand as they waited for their turn and they could not exit the OPD. Exiting the OPD meant that one could miss his/ her turn of seeing the doctor and as such participants will prefer standing to wait for their turns even when the OPD was full. To this end, coming to the hospital was noted to be a least preference for participants as they were likely to spend an entire day in pursuit of receiving health care. A contributing factor to long waiting hours at the hospital was identified to be inadequate number of staff (notably doctors) at the OPD. However, the noted difficulties associated with accessing hospital services made participants patronise herbal medicines as the herbal practitioners and their services were readily accessible in the community:

*“In the hospital, if you don’t have time then you do not have to even go. I get hungry but I cannot go and eat because the nurses might call me to see the doctor when I leave. If I miss that, it will be difficult getting me to see the doctor so I have to wait with the hunger. Sometime ago, I went there with cough complaints everyone looked at me when I cough so I thought the nurses would even allow me to see the doctor and leave for the house but it was some kind of first, come first served thing.” (Female, 50–59 years)*

*“Most of the time, I have to wait for long hours to see the doctor. Sometimes we are informed there are only two doctors here and so we have to be patient to see them one after the other. After this queue, I will have to get medications at the pharmacy and that is also another long queue” (Female, 60-69 years)*

*“Things are faster with the herbalist at the community center. There is nothing like retrieving folders or going to lab. I talk with him and he gives me medication and I am off” (Female, 70-79 years)*

Also, it was identified that older persons required information regarding their health status and the progress they have made in meeting treatment goals. However, in some cases participants could not obtain this information and that made them uncertain about how they are progressing. This enabled some participants to utilise herbal preparations as the herbal practitioners provided information about their conditions and offered encouragement to continue on the herbs even when no positive effects were noted by participants. This may reflect that relationship among older persons and health care providers played a part whilst accessing health services:

*“Sometimes I wish to spend more time talking with the doctor so that I can ask more questions but because other patients are waiting, I have to hurry and leave but I have questions bothering me.” (Female, 60–69 years)*

*“The herbalist has time to talk with me and assure me I will get better so I will keep coming here” (Female, 50-59 years)*

In addition to the above, it was noted that readily available medications or herbs further enabled older persons utilise a particular health care service. Older persons who utilised hospital services noted that medications were not always available and have to visit several pharmacies. In some cases, the medications were not actually available. In other instances, the medication was available but expensive and as such the older person could not afford it. However, older persons who patronised herbal preparations noted that herbs were readily available and easy to use as well:

*“I can easily pluck the herbs in the forest for free” (Male, 60–69 years).*

*“Sometimes too, the medicines are not even available in the hospital and you have roam in the town searching for the drug. If I go to one or two places and I cannot get the drug, I just stop searching for the drug and stay on the herbs till it is time for the monthly review.” (Female, 70–79 years).*

##### Health care cost

The cost associated with health service utilisation was also identified as a factor that enabled older persons utilise health services. With the existence of the National Health Insurance Scheme, older persons were required to make cash payments for services not covered by the scheme. Thus, utilising health care services at the hospital meant having enough money to pay for the services rendered. The cost associated with hospital service utilisation was described as expensive by participants. In the presence of non-availability of money, older persons went to the extent of borrowing money to cater for their health care needs. Despite the cost of services, participants expressed the need to be totally free of their ailments but this appeared unachievable as the conditions were chronic in nature. To this end, participants noted that the health insurance scheme (NHIS) is not supportive as it offered limited help with regards to covering health care costs:

*“Coming to the hospital is expensive. When the NHIS came at first, I was told most of the things in the hospital were free but now I have to pay for almost everything. I don’t know why it is like that but the president should do something about it. I am a farmer and if my products are not sold, it will be difficult to get money to come here” (Male, 50–59 years)*

However unavailability of money implied absence at the hospital for review. In some instances, they utilised part of the entire service available:

*“hmmmmm……… I have said from the beginning that it is expensive coming here and when I do not have money, I do not even come at all. Even with the health insurance, the laboratory test I have to do cost me 16 Ghana cedis. As for the medication, the insurance covers only one and I have to buy the other ones. Sometimes I keep the prescriptions till the following month when I have money to purchase them. It is a problem for me (caps chin in the right palm)” (Female, 50–59 years)*

*“The NHIS used to be very helpful because it is covered almost everything in the country. Previously, I did not pay anything when I came to the hospital but now even the medications that I used to get for free with the NHIS, I have to pay for it. Going to the lab to check my sugar level too comes with costs now and that increases the amount of money I spend whenever I come here. Am sure you now understand why I said if I don’t have money, I will not even try going there” (Male, 60-69 years)*

At some points, older persons missed medical appointments because of unavailability of money even when they were registered with the NHIS. However, costs associated with traditional medicine services were noted to be cheaper as compared to services offered at the hospital and in some cases, participants received free services from the traditional practitioners. These enabled older persons to utilise their services especially when they had limited financial resources:

*“I am supposed to go for check-up every month but I cannot keep to it because coming here is preparation so if I have money, I will come but sometimes the costs of the medications and services are so high that I cannot afford so I stay home” (Male, 50–59 years)*

*“I even like the herbs because it is not for sale; I only give a small token to the herbalist and then I have the herbs. If not for my daughter, I will see the herbalist every time instead of going to the hospital” (Female, 60-69 years).*

## Discussion

According to Andersen [15] individuals must first perceive illness or the likelihood of its occurrence for the use of health services to occur. In this study, the medical condition (symptom and diagnosis) experienced by participants made them require health care. This finding corroborates with those by Kohno et al., [11] as they noted that need determinants associated with Japanese retirees utilising health services in Malaysia included perceived health need by the older person, medical symptoms and self-rated general health status. In addition to existence of symptoms, the current study noted that these symptoms interfered with the older persons’ daily activities and as such they felt the need to return to their previous state of health: a finding that was not identified in previous studies and this could serve as an explanation as to why medical symptoms serve as a need factor.

Furthermore, Kim and Lee [21] and Exavery et al., [12] identified existence of chronic illness as a need factor as there was a need for continuing contact with health professionals (professional evaluation). In similar lines, the current study noted that by seeking professional evaluation for their illnesses, participants were made to come for reviews monthly, two monthly or three monthly. Thus, chronic illness required on-going health utilisation so as to monitor the progress of older persons and maintain good health.

Andersen et al., [13] have described predisposing determinants as those characteristics that make some persons utilise health services more than others. A theme captured in this study was perceived effects of ageing. Participants felt that as they grew older, they became ill more often and that made them require utilisation of outpatient services. Though Kim and Lee [21] noted that increasing age was associated with a lower outpatient service usage, the current study identified that increasing age predisposed an older person to utilise those services. This is because participants’ associated advancing age with physical symptoms such as joint pains may require more outpatient services and it substantiates the findings of Jahangir et al., [14] as they noted that increasing age was positively associated with increased use of preventive services which were available on outpatient basis. This could mean that preventive services played a major role in the health of an older adult and as such policy may need to consider increasing those services.

Furthermore, beliefs and past health care experiences were also identified to predispose older persons to utilise health services. An aspect of the findings substantiate those of Wong and Diaz [22] in that if health conditions in the past predisposed the older person to utilise health care services more and they were subsequently satisfied, they were likely to use the services again. However, a unique sub-theme which emerged from the current study was that beliefs related to the aetiology of a particular disease further predisposed an older person to utilise either orthodox or traditional services. According to Andersen [15] health beliefs include a wide range of personal thinking and behaviours such as attitudes, values and knowledge that people develop throughout their lives, pertaining to healthcare services. Andersen and Davidson [23] further argue that health beliefs influence the way people formulate ideas about their own need for healthcare services. In relation, this may mean that participants might think that some conditions may require one to visit the hospital whilst for other conditions, it may be inappropriate to visit the hospital. This assertion may reflect their past encounters with the health system. If the health service was successful in achieving optimum outcomes, the older person might want to utilise it again. However, if this did not occur and the disease was attributed to supernatural causes, traditional medicines were utilised. This specifies that both orthodox and traditional services at a particular time met a specific need of an older person. Thus, a better collaboration between these modes of service delivery may be helpful and this may require policy support.

Even though individuals may be predisposed to use health care services, some means need to be available to ensure their actual usage. These factors have been described as enabling determinants [13]. For this study, enabling factors were identified as family influence and support, accessibility and health care costs.

Health care costs were generally noted to be expensive by participants in this study even though they had all registered with the NHIS. This is because the Insurance Scheme which has been identified by Wong and Diaz [22] as an enabling factor is unable to meet the entire cost of health utilisation services by older persons. This implies that lack of adequate finances to supplement the NHIS may serve as a barrier to health care and as such participants either utilised traditional medicines or delayed their attendance to the hospital. This confirms the findings of Kohno et al., [11] as they noted that lack of enabling resources may delay the health care utilisation by older persons. Unique to this current study was that as participants delayed in utilising health care, they utilised traditional medicines as a substitute. However, this study could not establish the outcomes associated with this utilisation.

In meeting health care costs not covered by the NHIS, it was identified from this study that family support played an essential role. This is because older persons with family support were able to make it to the hospital for reviews and participants without support missed medical appointment and used herbal preparations more frequently. This finding supports an earlier finding by Jahangir et al., [14] as they identified the existence of family income as an enabling factor.

Aside finance, accessibility was also noted as an enabling factor. Accessibility was noted at four levels in this study and appeared to dominate most part of the interview: accessibility to the health facility, health professional, medications and information regarding their health status. Though some participants lived close to the health facility, others had to travel to the facility; in which case their first point of utilisation was the pharmacy. On some occasions, transport fares presented a challenge to older persons as that implied that they needed extra money to pay for the fares. Along similar lines, Exavery et al., [12] noted that ability to be transported to the health facility played a role in enabling older persons utilise health services. Though Parmar et al., [24] have noted that living far from the hospital affected the enrolment of older persons into the insurance scheme, it was identified that all participants in this study have registered with the NHIS. This variation could be related to the fact that exemption of older persons from paying premiums in Ghana has attracted greater enrolments.

In terms of accessibility to the health professional, participants noted that it was time consuming with cumbersome processes. These findings are in line with those indicated by Rooy et al., [25] as they reported that long waiting times affected older persons’ utilisation of hospital services and resulted in them utilising herbal products as these were readily available in the communities. Similar findings were also reported by Aboderin [26] and Goins et al., [27]. This may mean that older persons wanted to be attended to by health professionals as soon as they came to the hospital but in most cases, they did not experience that. Thus, there may be a need to improve the staff strength at the facility to enhance rapid delivery of services to patients. In addition there may be a need to encourage health professionals (nurses and physicians) to consider specialising in geriatrics so as to offer specialist care to older persons who visit the facility.

In terms of medications, participants noted that they sometimes had to roam in search of them as some types of medications were unavailable in the hospital. In some instances, the medications were available but expensive in which case an older person either utilised traditional medicine or saved money to purchase the medications at a later date. In similar lines, Etowa et al., [28] have asserted that older persons usually utilised self-medication and traditional preparations as these were cheaper and easily accessible. This might represent an avenue for policy consideration so as to foster closer working relationship between orthodox and traditional services.

Participants expressed the need to have information regarding how well they were progressing but in most cases, they could not access this information and that made them uncertain. Participants who visited the traditional herbal practitioners noted that they were available to provide information which was basically explanations of the symptoms an older person was experiencing. In similar lines, Rooy et al., [25] noted that even though older persons appreciated modern health care services, they utilised herbal products due to the limited number of health care staff and an increasing health care provider to patient ratio at the hospital.

As this study was conducted in a district hospital, a similar study may be carried out in a teaching hospital to compare findings as it is expected that the latter may have more specialised forms of services for all patients and as such variations in their experiences. As participants noted utilisation costs to be high, future research might consider actual costs associated with health service utilisation in comparison with those covered by the NHIS so as to enable more objective assessment of the insurance scheme and make further recommendations.

Findings from this study are however limited and unique to the setting in which it was undertaken. Also the findings may be limited to older persons utilising discretionary health service (outpatient hospital services) as the study focused on them. Thus, other studies are warranted in exploring the phenomenon among older persons using other forms of health services in the district.

## Conclusion

Findings from this study have provided an understanding of older persons’ experiences with outpatient hospital services. Though older persons in the district may feel the need to utilise health services on outpatient basis, the enabling factors (notably finance) appeared to be a driving force to actual utilisation. Thus, more innovative health care financing strategies are needed to enhance the coverage of health services for older persons as even though they may feel the need to utilise the health services, financial constraints may cause a delay in the process. In addition, there may be a need to increase the staff strength so as to shorten the waiting time for older persons seeking health care as well as enhance easy accessibility to health professionals. In this regard, nurses and doctors may need to be supported to undertake specialist education in geriatrics so as to plan and execute programmes tailored to meet the needs of older persons seeking health care.

## References

[1] World Health Organization (2010). Action on the social determinants of health: learning from previous experiences.

[2] United Nations. Department of economic. (2010) ‘World population ageing 2009: Vol. 295. United Nations Publications.

[3] Kinsella KG, Phillips DR (2005). ‘Global aging: the challenge of success’, population reference bureau. Washington.

[4] Lloyd-Sherlock P (2002). Social policy and population ageing: challenges for north and south. Int J Epidemiol.

[5] Kwankye SO (2013). Growing old in Ghana: health and economic implication. Postgraduate Medical Journal of Ghana.

[6] Polder JJ, Bonneux L, Meerding WJ, Van der Maas PJ (2002). Age-specific increases in health care costs. Eur J Pub Health.

[7] Ghana Statistical Service, (2013) ‘2010 Population and Housing Census: National Analytic Report. http://www.statsghana.gov.gh/docfiles/2010phc/National Analytical Report.pdf (Accessed on 09/02/2016).

[8] Duodu Y (1998). The need for geriatric care services in Ghana. Southern African Journal of Gerontology.

[9] Ghana Public Health Association (2014) ‘Health of the Elderly and Policy Implications in Ghana. http://www.ghanaweb.com/GhanaHomePage/health/Health-of-the-elderly-and-policy-implications-in-Ghana-327185 (accessed on 10–03-2016).

[10] Tawiah EO (2011). Population ageing in Ghana: a profile and emerging issues. Afr Popul Stud.

[11] Kohno A, Farid NDN, Musa G, Aziz NA, Nakayama T, Dahlui M. ‘Factors affecting Japanese retirees' healthcare service utilisation in Malaysia: a qualitative study’. BMJ Open. 2016;6(3),P.e010668. 10.1136/bmjopen-2015-010668 PMC4809084 27006344

[12] Exavery A, Klipstein-Grobusch DC, Debpuur C. ‘Self-rated health and healthcare utilization among rural elderly Ghanaians in Kassena-Nankana District’. Working paper. Presented in session 59: trends, patterns, and consequences of non-communicable diseases in Africa. 6th Union for African Population Studies Conference (UAPS). 2011. Ghana: Navrongo Health Research Centre (NHRC); 2010.

[13] Andersen R. Health service use. National Trends and Variations. 1972:1953–71.

[14] Jahangir E, Irazola V, Rubinstein A. Need, enabling, predisposing, and behavioral determinants of access to preventative care in Argentina: analysis of the national survey of risk factors. PLoS One. 2012;7(9):1–6. 10.1371/journal.pone.0045053 PMC3440415 22984608

[15] Andersen R (1995). Revisiting the behavorial model and access to medical care: does it matter?. J Health Soc Behav.

[16] Burns N, Grove SK (2007). Understanding nursing research: building an evidence-based practice.

[17] Waldrop DP, Kramer DJ, Skretny JA, Milch RA, Finn W. Final transitions: family care giving at the end of life. J Palliat Med. 2005;8(3):623–38. 10.1089/jpm.2005.8.623 15992205

[18] Parahoo K (2006). Nursing research: principles, process and issues.

[19] Mason J. Qualitative Researching. SAGE: London; 2006 pp 62–83.

[20] Bryman A (2012). Social research methods.

[21] Kim HK, Lee M (2016). Factors associated with health services utilization between the years 2010 and 2012 in Korea: using Andersen's behavioral model. Osong public health and research perspectives.

[22] Wong R, Díaz JJ, Higgins M (2006). Health care use among elderly Mexicans in the United States and Mexico the role of health insurance. Research on Aging.

[23] Andersen RM, Davidson PL (1997). Ethnicity, aging, and oral health outcomes: a conceptual framework. Adv Dent Res.

[24] Parmar D, Williams G, Dkhimi F, Ndiaye A, Asante FA, Arhinful DK, Mladovsky P (2014). Enrolment of older people in social health protection programs in West Africa - does social exclusion play a part?. Social Science & Medicine.

[25] Rooy VG, Amadhila EM, Mufune P, Swartz L, Mannan H, MacLachlan M (2012). Perceived barriers to accessing health services among people with disabilities in rural northern Namibia. Disability & Society.

[26] Aboderin I. ‘Advancing health service provision for age-related non-communicable disease and older persons in Africa: identifying key information and training needs. African research on ageing network (AFRAN), policy-research dialogue. 2008.

[27] Goins RT, Williams KA, Carter MW, Spencer M, Solovieva T (2005). Perceived barriers to health care access among rural older adults: a qualitative study. J Rural Health.

[28] Etowa J, Wiens J, Barnard WT, Clow B (2007). Determinants of black women’s health in rural and remote communities. Can J Nurs Res.

